# Molecular Detection of Equine Herpesvirus Types 1 and 4 Infection in Healthy Horses in Isfahan Central and Shahrekord Southwest Regions, Iran

**DOI:** 10.1155/2015/917854

**Published:** 2015-09-01

**Authors:** Taghi Taktaz Hafshejani, Shahin Nekoei, Behnam Vazirian, Abbas Doosti, Faham Khamesipour, Madubuike Umunna Anyanwu

**Affiliations:** ^1^Department of Clinical Sciences, Faculty of Veterinary Medicine, Shahrekord Branch, Islamic Azad University, Shahrekord, Iran; ^2^Faculty of Veterinary Medicine, Shahrekord Branch, Islamic Azad University, Shahrekord, Iran; ^3^Biotechnology Research Center, Shahrekord Branch, Islamic Azad University, Shahrekord, Iran; ^4^Young Researchers and Elite Club, Shahrekord Branch, Islamic Azad University, Shahrekord, Iran; ^5^Microbiology Unit, Department of Veterinary Pathology and Microbiology, University of Nigeria, Nsukka, Enugu State, Nigeria

## Abstract

This study was undertaken to investigate molecularly the occurrence of EHV-1 and EHV-4 infection among equine population in regions, Iran. Blood samples from 53 and 37 randomly selected horses settled in Isfahan and Shahrekord, Iran, respectively, were collected. Detection of EHV-1 and EHV-4 genes in the blood samples was done using polymerase chain reaction (PCR). Out of 53 and 37 samples from Isfahan and Shahrekord, 4 (18.18%) and 3 (8.10%) were positive for PCR of EHV-1, respectively. Nine (16.98%) and 6 (16.21%) were positive for PCR of EHV-4, while 6 (11.32%) and 3 (8.10%) were positive for PCR of both EHV-1 and EHV-4, in Isfahan and Shahrekord, respectively. Of the 7 blood samples positive for EHV-1, 4 (16.66%) and 3 (8.10%) were from horses >3 years old while 2 (18.18%) and 1 (16.66%) were from 2-3 years old horses, in Isfahan and Shahrekord, respectively. Out of the 7 and 3 samples positive for PCR of EHV-1 in Isfahan and Shahrekord, 4 (22.2%) and 1 (7.69%) were Standardbred, while 3 (14.28%) and 2 (13.33%) were Thoroughbreds, respectively. EHV-4 was detected in blood of 4 (22.22%) and 2 (15.83%) Standardbreds and from 4 (19.04%) and 4 (26.66%) Thoroughbred horses in Isfahan and Shahrekord, respectively. This study has shown that horses settled in Isfahan central and Shahrekord southwest regions, Iran, are infected by EHV-1 and EHV-4 and thus serve as potential reservoirs and disseminators of the viruses.

## 1. Introduction

Equine herpesvirus type 1 (EHV-1) also called “equine abortion virus” and equine herpesvirus type 4 (EHV-4) also called “equine rhinopneumonitis virus” are linear double-stranded DNA viruses which belong to the family Herpesviridae of the genus* Varicellovirus* in the subfamily Alphaherpesvirinae [1, 2]. These viruses are ubiquitous and infection of horses by them is among the most important conditions in equine industry worldwide [2, 3]. Recent outbreaks of EHV-1 and EHV-4 infection in some parts of the world aroused public interest, which led to it being tagged as an emerging threat [4]. Infections by EHV-1 and EHV-4 are responsible for huge economic loss in equine industry [5–8] where they have been incriminated in cases of abortions and perinatal mortality, neonatal mortality, respiratory and neurologic diseases [8–10]. Economic impact of infection by the viruses in equine population occurs in 3-fold: respiratory disease interrupting athletic programs, abortions resulting in loss of replacement stock and dissemination of viruses in environment, and neurological disease (equine herpes myeloencephalopathy (EHM)) resulting in suffering, loss of life, and extensive movement restrictions which consequently disrupts breeding or training schedules, causing management difficulties at training centers, racetracks, and horse events [2, 4]. Epidemiological studies showed that EHV-1 and EHV-4 infections could be latent (causing no clinical condition and no clinical sign) in an infected horse due to absence of virus in peripheral blood mononuclear cells [3, 10]. Horses with latent and/or active infection harbour these viruses and serve as carriers and reservoirs of infections [7, 10, 11]. These carriers become disseminators of the viruses when they are stressed (transported, raced, starved, etc.) and immunocompromised [2–4]. When horses are infected by the viruses and they show clinical signs, the severity is often influenced by factors such as age, physical condition of the host, type of infection (i.e., whether primary activation, secondary activation, or a reactivation of a latent virus), immune status of the host, and the virulence of the strain involved [8]. Breed and sex may also affect the rate and severity of infection by the viruses [4]. Spread of the viruses among horses is fast via nasal inhalation of aerosol droplets and/or direct contact [2, 8, 9, 12]. The viruses are spread easily when horses are in close proximity in stables, during transportation, race competitions, and breeding [10]. These factors are often unavoidable in countries with large equine population including Iran. Zoonotic infection by these viruses especially EHV-1 is recognized [2].

Because EHV-1 and EHV-4 infections are often latent and the diseases characterized by nonpathognomonic (nonspecific) clinical signs [10], detection of infections especially in clinically healthy horses, and/or diagnosis of the diseases are usually difficult [3], detection of infections by the viruses in blood by virus isolation, direct immunofluorescence, and immunohistochemical (IHC) method is often difficult [8]. Polymerase chain reaction (PCR) has been widely proved to be a quick, very sensitive, and reliable method for detection of infection by EHV-1 and EHV-4 [8–10, 13–16]. However, detection of the viruses in blood indicates viraemia resulting from active infection, and latent infection alone may not give a positive PCR test using blood sample [4].

Reports on detection of equine herpesvirus (EHV) from clinical samples focused mainly on EHV-1 [4, 17–19]. This led to a long-held speculation that EHV-1 infection may be more common than EHV-4 infection. This necessitated surveillance studies to detect EHV-1 among equine populations in different parts of the world such as North America [20] and South America [8, 17, 18, 21]. Reports on the occurrence of EHV-4 infection in equine population are rather scanty. Studies in countries such as America [3], Colombia [16], and Egypt [22] revealed higher prevalence of EHV-4 infection than EHV-1 infection among equine populations. In the available literature, two studies that detected EHV among equine population in Iran included the serological study of Momtaz and Hematzadeh [23] in Chaharmahal and Bakhtiari province in the southwestern part of the country and the molecular study of Sarani et al. [10] in northeastern region of the country. The studies reported EHV-1 detection rates of 39.08 and 0% among the sampled horses, respectively, while EHV-4 detection rate was reported to be 68.96 and 100%, respectively. Both results also suggested that EHV-4 infection may be more prevalent among equine populations in Iran. No study has been conducted to detect neither EHV-1 nor EHV-4 infection among equine population in Isfahan, central region of Iran, whereas there are many horse herders in the region. Moreso, the central location of Isfahan, makes it a convergent point for horses from other regions of the country, for horse race competitions, sales, and so forth. These factors may encourage the spread of equine herpesviruses among horses in the region and beyond, if they harbour these viruses undetected. Due to proximity, easy transmission of the viruses from infected horses in Chaharmahal and Bakhtiari province to those in Shahrekord (the capital of the province) and* vice versa* could often occur. But no study has been conducted to detect these viruses in horses settled in Shahrekord. Studies showed that molecular detection of EHV infection is more accurate and reliable owing to the limitations of serological test [10, 24]. Therefore, there is need to detect the presence of the viruses among equine population in Isfahan central and Shahrekord southwest regions, Iran. Detection of infection by EHV-1 and EHV-4 viruses is crucial for the control of transmission of the viruses and treatment of infected horses [25]. The objective of this study, therefore, was to detect molecularly EHV-1 and/or EHV-4 infection among apparently healthy equine population in Isfahan central and Shahrekord southwest regions of Iran.

## 2. Materials and Methods

### 2.1. Sampling

This cross-sectional study was conducted between February and December, 2014. A total of 53 and 37 horses settled in Isfahan central and Shahrekord southwest regions, Iran, respectively, were randomly selected. The breed, sex, and age of each of the horses were noted and appropriately recorded. Blood sample was collected by venipuncture from the external jugular vein from each of the horses using anticoagulant (ethylenediaminetetraacetic acid (EDTA)) containing vacutainer. The samples were transported aseptically in ice packs to the Biotechnology Research Center of Islamic Azad University, Shahrekord, Iran, and stored at −20°C until needed.

### 2.2. Detection of EHV-1 and EHV-4 Genes in Blood Samples

Viral genomic DNA in the blood samples was extracted using DNA extraction kit (Cinnagen, Tehran, Iran) following the manufacturer's instruction. Concentration of extracted DNA from each blood sample was measured spectrophotometrically at 260 nm optical density following the method described by Sambrook and Russell [26]. Extracted DNA samples were kept frozen at −70°C until needed. Detection of extracted viral DNA as EHV-1 and/or EHV-4 gene was done by polymerase chain reaction (PCR) using specific primers and annealing temperature previously described (Table 1). Positive controls from the collection of the Biotechnology Research Centre, Islamic Azad University, Iran, were included in each PCR reaction, while sterile distilled water was used as the negative controls. The amplification of* EHV-1 *and* EHV-4* DNA was done using thermocycler (Eppendorf, Hamburg, Germany). PCR reaction for* EHV-1 *was performed as follows (30 cycles): denaturation at 94°C for 60 s, annealing at 65°C for 60 s, extension at 72°C for 60 s, and then final incubation at 72°C for 7 min. PCR reaction for* EHV-4 *was performed as follows (33 cycles): denaturation at 94°C for 60 s, annealing at 66°C for 60 s, extension at 72°C for 60 s, and then final incubation at 72°C for 5 min. Analysis of the PCR products was performed in 1.5% horizontal agarose gel electrophoresis stained with ethidium bromide under UV light. The PCR products were identified by 100-base pair (bp) DNA size marker (Fermentas, Germany).

## 3. Results

### 3.1. Occurrence of EHV-1 and EHV-4 among Equine Population in Isfahan and Shahrekord 

**Figure 1:**
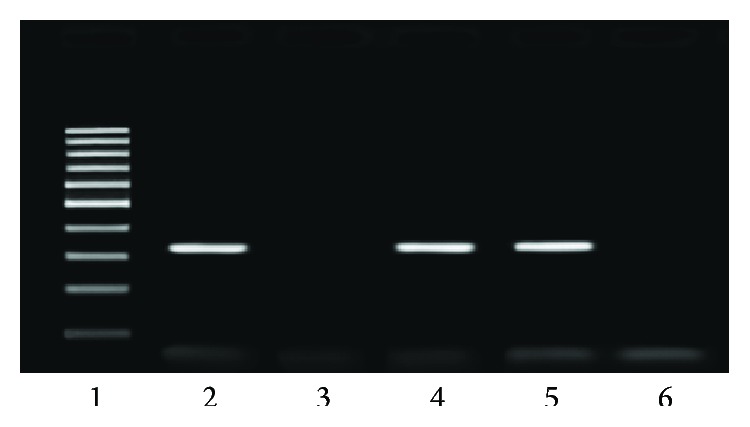
Ethidium bromide-stained 1.5% agarose gel electrophoresis of PCR products for equine herpesvirus type 1 (EHV-1). Lane 1: 100-base pair DNA marker; lane 2: positive control; lane 3: negative control (distilled water); lanes 4 and 5: positive samples; lane 6: negative sample.

**Figure 2:**
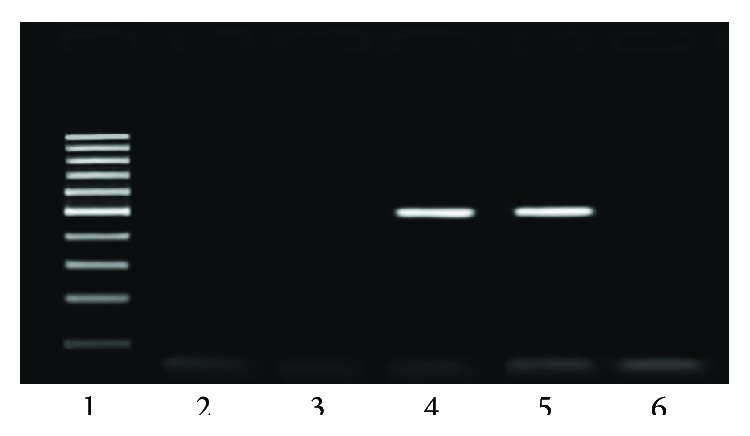
Ethidium bromide-stained 1.5% agarose gel electrophoresis of PCR products for equine herpesvirus type 4 (EHV-4). Lane 1: 100-base pair DNA marker; lane 2: negative control (distilled water); lanes 3 and 6: negative samples; lane 4: positive samples; lane 5: positive control.

Out of 53 samples from Isfahan, 7 (13.20%) were positive for PCR of EHV-1 and 9 (16.98%) for PCR of EHV-4 while 6 (11.32%) were positive for PCR of both EHV-1 and EHV-4 (Table 2, Figures 1 and 2). Out of 37 samples from Shahrekord, 3 (8.10%) were positive for PCR of EHV-1 and 3 for PCR of both EHV-1 and EHV-4 while 6 (16.21%) were positive for PCR of EHV-4.

### 3.2. Occurrence of EHV-1 and EHV-4 Infections among Different Age Groups of Horses in Isfahan and Shahrekord 

Of the 7 blood samples positive for PCR of EHV-1 in Isfahan, 1 (9.09%) was obtained from a horse 1-2 years old and 2 (18.18%) were obtained from horses 2-3 years old while 4 (16.66%) were collected from horses >3 years old (Table 3). Out of the 9 blood samples positive for PCR of EHV-4 in Isfahan, 4 (36.36%) were collected from horses 2-3 years old while 5 (20.83%) were obtained from horses >3 years old. Out of the 6 blood samples positive for PCR of both EHV-1 and EHV-4 in Isfahan, 2 (18.18%) were 2-3 years old while 4 (16.66) were >3 years old. None of the blood samples from horses <1 year old was positive for PCR of neither EHV-1 nor EHV-4.

In Shahrekord, of the 3 blood samples positive for PCR of EHV-1, 1 (16.66%) was obtained from a horse 2-3 years old while 2 (8.33%) were collected from horses >3 years old (Table 3). Out of the 6 blood samples positive for PCR of EHV-4, 1 (25%) was collected from a horse 1-2 years old, while 4 (16.66%) were obtained from horses >3 years old. Out of the 3 blood samples positive for PCR of both EHV-1 and EHV-4, 1 (16.66%) was from a horse 2-3 years old while 2 (8.33%) were from horses >3 years old. None of the blood samples from horses <1 year and 1-2 years old was positive for PCR of EHV-1.

### 3.3. Occurrence of EHV-1 and EHV-4 Infections in Different Horse Breeds in Isfahan and Shahrekord

In Isfahan, out of the 7 blood samples positive for PCR of EHV-1, 4 (22.22%) were obtained from horses belonging to the Standardbred breed while 3 (14.28%) were from horses which belonged to the Thoroughbred breed (Table 4). Out of the 9 blood samples positive for EHV-4, 4 (22.22%) were collected from horses which belonged to Standardbred and Thoroughbred breeds while 1 (14.28%) was collected from a horse of the Arab breed. Of the 6 blood samples positive for PCR of both EHV-1 and EHV-4, 3 (16.66%) were from horses of the Standardbred and 3 (14.28%) were from horses of the Thoroughbred breeds. None of the blood samples obtained from horses of the Turkoman breed was positive for neither EHV-1 nor EHV-4.

In Shahrekord, out of the 3 blood samples positive for PCR of EHV-1, 1 (7.69%) was collected from a horse belonging to the Standardbred breed while 2 (13.33%) were from horses belonging to the Thoroughbred breed (Table 4). Out of the 6 blood samples positive for EHV-4, 2 (15.38%) were collected from horses which belonged to Standardbred while 4 (26.66%) were from Thoroughbred breeds. Of the 3 blood samples positive for PCR of both EHV-1 and EHV-4, 1 (7.69%) was from a horse of the Standardbred while 2 (13.33%) were from Thoroughbred. None of the blood samples collected from the Arab and Turkoman breeds was positive for neither EHV-1 nor EHV-4.

## 4. Discussion 

In this study, EHV-1 and EHV-4 infections in apparently healthy horses settled in Isfahan central and Shahrekord southwest regions, Iran, were detected molecularly using polymerase chain reaction (PCR) method. The fact that blood samples from some of the horses were positive for PCR of the viruses is indicative of viraemia and thus suggestive of active infection by the viruses in the horses [4]. Absence of clinical signs in all the sampled horses in this study could be attributed to the ability of their immune system to suppress most of the viruses keeping them in a latent state with few detectable viruses in the peripheral blood mononuclear cells (PMBCs) [3, 24, 27]. The equine herpesvirus (EHV) detection rate among horses in Isfahan suggests that spread of the viruses among equine population in Isfahan is of higher rate than those in Shahrekord. These findings suggest that the infected horses are carriers of EHV-1 and EHV-4 and could serve as sources of infection (following stress, immune suppression, and virus shedding) to other horses within and outside the study areas [3]. The source(s) of the viruses could be from apparently healthy and/or nonhealthy in-contact carriers. These carriers could have transmitted the viruses to the sampled horses during transportation, training periods, race competitions, or breeding [2, 10]. It is also possible that the horse keepers, jockeys, or animal health workers transmitted the viruses from infected horses to the sampled horses by direct contact during grooming, riding, or medical examinations/treatment [2]. Both detection rates in Isfahan (EHV-1: 13.20%, EHV-4: 16.98%) and Shahrekord (EHV-1: 8.10%, EHV-4: 16.21%) observed in this study are lower when compared with 88% EHV detection rate reported by Sarani et al. [10] among equine population in northeast of Iran. Variation in detection rates among equine population in these study areas could be due to the differences in the rate of exposure to infection, samples analysed, immune status of the horses, season, and breed of the horses.

The result of Isfahan may suggest that the stallions (EHV-1: 18.18%, EHV-4: 22.72%) were infected more than the mares (EHV-1: 9.67%, EHV-4: 12.90%) and therefore had more active infection by the viruses. But the result of Shahrekord (EHV-1: stallion (8.96%), mare (7.14); EHV-4: stallion (8.69%), mare (28.57%)) suggested otherwise. This finding suggests that sex may not have played a role in EHV infection. Momtaz and Hematzadeh [23] reported that sex did not significantly affect infection of horses in Chaharmahal and Bakhtiari province, Iran. Other authors elsewhere had reported that sex is a factor in the epidemiology of infection by EHV-1 and EHV-4 [4, 28]. Nevertheless, the higher EHV detection rate in stallions in Isfahan could be a result of their sexual activities such as seeking for mating partners (during which they tend to sniff with their nose the nostrils and vulva of other horses), their frequent use for breeding programmes (where one stallion could be used to breed many mares), races, and competitions more than the mares. These factors could have predisposed and exposed these stallions to EHV infections more than the mares [4]. This higher detection among stallions in Isfahan contrasts Goehring et al.'s [28] who reported higher infection rate among mares in Netherlands. The differences in infection rate among sexes in these studies may be due to differences in rate of exposure to infection, health status (such as pregnancy and suckling in mares), age, previous vaccinations, or immune status of the horses sampled in the study areas.

The fact that the gene of EHV-4 was detected in 9 (16.98%) and 6 (16.21%) of blood samples from Isfahan and Shahrekord, respectively, against 7 (13.20%) and 3 (8.10%) samples positive for EHV-1 suggests that EHV-4 infection is more prevalent than the EHV-1 infection among equine population in both Isfahan and Shahrekord, Iran. These findings agree with the reports of Momtaz and Hematzadeh [23] and Sarani et al. [10] that EHV-4 infection seems to be more prevalent than EHV-1 infection among equine populations in Iran. Detection of genes of both EHV-1 and EHV-4 in blood samples of horses in Isfahan and Shahrekord suggests the occurrence of mixed infection by both viruses in horses in the study areas. Interestingly, despite the mixed infection, the horses were clinically healthy. This suggests that horses concurrently infected with EHV-1 and EHV-4 could harbour both viruses simultaneously in a latent state. This finding suggests that the animals were immunocompetent for them not to have manifested any clinical sign. It could also be that the horses have not been subjected to stressful events (poor housing, transportation, competitions, physiological stress, e.g., pregnancy, etc.) which would have reactivated viruses in latency [3, 29]. The 16.98 and 16.21% EHV-4 detection rates in Isfahan and Shahrekord, respectively, are lower than 68.96% EHV-4 serological detection and 88% EHV-4 molecular detection rates reported by Momtaz and Hematzadeh [23] and Sarani et al. [10] among equine population in the southwestern and northeastern part of Iran, respectively. Momtaz and Hematzadeh [23] and Ohta et al. [25] reported 39.08 and 33.3% EHV-1 serological and molecular detection rates in southwest Iran and Japan, respectively. These rates are higher than the 13.20 and 8.10% EHV-1 detection rates in Isfahan and Shahrekord, respectively, recorded in this study. Sarani et al. [10] did not detect EHV-1 among equine population in northeast Iran. The lower EHV-1 and EHV-4 infection rates in this study may be due to strain variability or due to latency of infection and hence very low dose of the viruses was present in the blood [10]. It could also be that horses in the other study areas were more exposed to infection than those in this present study. Cross-reactivity of antibodies against the viruses [3, 10] may also account for the higher EHV-1 and EHV-4 detection rates reported by Momtaz and Hematzadeh [23] than the rates recorded in this study. However, the higher EHV-1 detection rates in both Isfahan and Shahrekord against that of Sarani et al. [10] in northeast Iran could be due to maternal immunity or previous vaccinations that resulted in production of antibodies which could have destroyed most of the viruses in the blood of the horses, hence a lower latency, active infection, and detection of the viruses in the previous study [2].

Age has been reported to be a factor that can influence infection by EHV-1 and EHV-4 [4]. In this study, EHV-1 gene was predominantly detected in the blood of horses >3 years old (16.66%) and 2-3 years old (16.66%) in Isfahan and Shahrekord, respectively, whereas none of the blood samples from horses <1 year old tested positive for PCR of EHV-1 in the study areas. These results suggest higher occurrence of EHV-1 infection in older/adult horses among horses in the study areas. This is further buttressed by the fact that while 1 (9.09%) blood sample from a horse 1-2 years old in Isfahan was positive for PCR of EHV-1, none (0%) was positive for it in Shahrekord. The higher detection rate in older horses in this study could be attributed to the fact that while the foals are usually kept with their suckling dams, unsold and unused for competitions, the adult horses are often transported in large numbers for race competitions and/or sales. These factors could have predisposed the older/adult horses to EHV-1 infection more than the foals. It could also be that maternally derived antibodies protected the foals and hence the lesser EHV-1 detection rate observed amongst them [2]. Detection of EHV-4 gene in the blood samples from 4 (36.36%) and 1 (16.66%) horses that are 2-3 years old in Isfahan and Shahrekord, respectively, and in those of 5 (20.83%) and 4 (16.66%) horses >3 years old suggests that EHV-4 infection also occurred more in the adult/older horses. This result further suggests that the adult horses could have been more exposed to infection by EHV-4 than the foals in both Isfahan and Shahrekord. This is further supported by the fact that, in both study areas, EHV-4 gene was not detected in blood samples from any of the horses <1 year old, and only 1 (16.66%) from a horse 1-2 years old in Shahrekord was positive for PCR of EHV-4. The higher detection rates among adult/older horses in this study corroborate the reports of Goehring et al. [28] and Henninger et al. [30] who reported higher EHV detection rates in horses >3 and 5 years old in Netherlands and North America, respectively.

Detection of EHV-1 gene in blood of 4 (22.22%) and 1 (7.69%) samples of horses of Standardbred breed and 3 (14.28%) and 2 (13.33%) samples of horses of Thoroughbred in Isfahan and Shahrekord, respectively, suggests that these breeds were more infected by EHV-1 than horses of the Turkoman and Arab breeds in which none of the viruses was detected in the study areas. Detection of EHV-4 in 22.22% Standardbred and 19.04% Thoroughbred in Isfahan and 15.38% and 26.66% Standardbred and Thoroughbred, respectively, in Shahrekord suggests that EHV-4 infection also occurred more in these breeds. These findings suggest that these breeds (Standardbred and Thoroughbred) could be more susceptible to infection by the viruses more than the Turkoman and Arab breeds of horses with 0–14.28% infection rates in the study areas. These results suggest a kind of variation in breed susceptibility to the viruses. Reports have suggested that certain horse breeds could be more susceptible to infection by EHV-1 and EHV-4 than others [4, 28]. Variation in immune status and rate of exposure to infection could account for the differences in the detection rates observed among the breeds. However, the higher detection rates among Standardbred and Thoroughbreds in this study could be because greater number of sampled stallions belonged to these breeds.

In conclusion, this study has shown that active and/or latent infection by EHV-1 and EHV-4 occurs among equine population in Isfahan central and Shahrekord southwest regions, Iran. These horses harbour the viruses and serve as their disseminators following stress and reactivation of latent infections. EHV-4 infection seems to be more prevalent than EHV-1 infection among equine population in the study areas.

## Figures and Tables

**Table 1:** Primer sequence used for detection of EHV-1 and EHV-4 genes in horses blood.

Virus	Primer sequence	Size (base pair)	Annealing temperature	GenBank accession numbers
EHV-1	F: 5′-GCAAACAACAGAGGGTCGATAGAAG-3′	342	65°C	JQ692316
	R: 5′-GTCGATGTCGTAAAACCTGAGAG-3′			

EHV-4	F: 5′-TATTGTTTCCGCCACTCTTGACG-3′	508	66°C	JX416462
	R: 5′-GTAGAATCGGAGGGCGTGAAGC-3′			

Key: EHV-1: equine herpesvirus type 1; EHV-4: equine herpesvirus type 4.

**Table 2:** Detection rate of EHV-1 and EHV-4 among equine population in Isfahan and Shahrekord.

Sex	Number of samples collected		Number (%) of horses infected with virus					
	Isf.	Sha.	EHV-1		EHV-4		Both EHV-1 and EHV-4	
			Isf.	Sha.	Isf.	Sha.	Isf.	Sha.
Stallion	22	23	4 (18.18)	2 (8.69)	5 (22.72)	2 (8.69)	4 (18.18)	2 (8.69)
Mare	31	14	3 (9.67)	1 (7.14)	4 (12.90)	4 (28.57)	2 (6.45)	1 (7.14)
Total	53	37	7 (13.20)	3 (8.10)	9 (16.98)	6 (16.21)	6 (11.32)	3 (8.10)

Keys: EHV: equine herpesvirus; EHV-1: equine herpesvirus type 1; EHV-4: equine herpesvirus type 4; Isf.: Isfahan; Sha.: Shahrekord.

**Table 3:** Occurrence of EHV-1 and EHV-4 infections among different age groups of horses in Isfahan and Shahrekord.

Age (years)	Number of samples collected		Number (%) of horses infected with virus					
	Isf.	Sha.	EHV-1		EHV-4		Both EHV-1 and EHV-4	
			Isf.	Sha.	Isf.	Sha.	Isf.	Sha.
<1	7	3	0 (0)	0 (0)	0 (0)	0 (0)	0 (0)	0 (0)
1-2	11	4	1 (9.09)	0 (0)	0 (0)	1 (25)	0 (0)	0 (0)
2-3	11	6	2 (18.18)	1 (16.66)	4 (36.36)	1 (16.66)	2 (18.18)	1 (16.66)
>3	24	24	4 (16.66)	2 (8.33)	5 (20.83)	4 (16.66)	4 (16.66)	2 (8.33)
Total	53	37	7 (13.20)	3 (8.10)	9 (16.98)	6 (16.21)	6 (11.32)	3 (8.10)

Keys: EHV-1: equine herpesvirus type 1; EHV-4: equine herpesvirus type 4; Isf.: Isfahan; Sha.: Shahrekord.

**Table 4:** Occurrence of EHV-1 and EHV-4 infections in different horse breeds in Isfahan and Shahrekord.

Breed	Number of samples collected		Number (%) of horses infected with virus					
	Isf.	Sha.	EHV-1		EHV-4		Both EHV-1 and EHV-4	
			Isf.	Sha.	Isf.	Sha.	Isf.	Sha.
Standardbred	18	13	4 (22.22)	1 (7.69)	4 (22.22)	2 (15.38)	3 (16.66)	1 (7.69)
Thoroughbred	21	15	3 (14.28)	2 (13.33)	4 (19.04)	4 (26.66)	3 (14.28)	2 (13.33)
Arab	7	7	0 (0)	0 (0)	1 (14.28)	0 (0)	0 (0)	0 (0)
Turkoman	7	2	0 (0)	0 (0)	0 (0)	0 (0)	0 (0)	0 (0)
Total	53	37	7 (13.20)	3 (8.10)	9 (16.98)	6 (16.21)	6 (11.32)	3 (8.10)

Keys: EHV-1: equine herpesvirus type 1; EHV-4: equine herpesvirus type 4; Isf.: Isfahan; Sha.: Shahrekord.
